# *Piriformospora indica* culture filtrate application adds brilliance to the promoting effects of facility warming on winter jujube fruit ripening

**DOI:** 10.1016/j.fochx.2024.101986

**Published:** 2024-11-09

**Authors:** Anping Shao, Junqiang Yang, Hao Li, Ruide Li, Yang Hu, Chunzhen Cheng

**Affiliations:** College of Horticulture, Shanxi Agricultural University, Taigu 030801, China

**Keywords:** Facility warming, High temperature, Winter jujube (*Ziziphus jujuba* mill. cv. Dongzao), Fruit ripening and quality, *Piriformospora indica*

## Abstract

Facility warming can promote fruit ripening of winter jujube (*Zizyphus jujuba* Mill. cv. Dongzao, DZ) for pre-marketing. Here, we compared the fruit development- and quality-related parameters of facility cultivated DZ trees grown at maximum temperature of 37 °C, 39.5 °C and 42 °C with and without *Piriformospora indica* culture filtrate (CF) treatment. Results showed that facility high temperature (HT) promoted DZ fruit enlargement in the early stage, accelerated fruit coloration and softening, and improved most fruit quality-related parameters. However, it decreased the vitamin C (VC) content and antioxidant capacities of DZ fruits, and eventually led to smaller fruits. CF treatment improved the positive effects of HT on DZ fruits and anthocyanins accumulation in fruit peel, and alleviated VC content reduction caused by HT. Our study demonstrated the promoting effects of facility warming and CF on fruit ripening and quality of winter jujube and revealed the underlying physiological mechanism.

## Highlights

• Facility high temperature (HT) can promote the fruit ripening of winter jujube (DZ).
• HT improved most fruit quality-related parameters, such as contents of total soluble solids, soluble sugars and so on.
• HT decreased the VC content and antioxidant capacities of DZ fruits.
• *Piriformospora indica* culture filtrate spraying can further improve the positive effects of HT on DZ fruits.

## 1 Introduction

Temperature, significantly affecting the growth and development of plants (Huang et al., 2022), is one of the most important environmental factors influencing crop production. In recent years, with the gradual increase of global temperature, the impact of high temperature (HT) on crop production has received increasing attentions. Evidences revealed that excessive HT will suppress photosynthesis and respiration abilities of plants, damage the cell membrane system (Zha et al., 2022), induce premature leaf senescence, decrease growth rates and pollen viability (Raju et al., 2020), and influence crop quality and yield (Xia et al., 2023). Moreover, as a double-edged sword, appropriate HT has been discovered to be beneficial to plant growth and crop production. In facility agriculture, heating facilities are commonly utilized. Researches demonstrated that appropriate temperature rise can shorten growth cycle (Sanwong et al., 2023), advance maturation (Arrizabalaga-Arriazu et al., 2020), and increase yield and quality of crops (Wang et al., 2024; Zhu et al., 2023). Therefore, the rational use of HT is recognized as an important mean for improving crop production and quality.

Jujube (*Ziziphus jujuba* Mill.), one of the oldest fruit trees, has been cultivated for more than 7000 years (Yang, Shen, et al., 2023). Its fruit production ranked the first among all dry fruits. Winter jujube (*Z. jujuba* Mill. cv. Dongzao, DZ) is a major fresh-eating jujube variety primarily cultivated in Shandong, Shanxi, Shaanxi, and Hebei provinces of China (Liu et al., 2020). Its fruits are loved by consumers for their delicate appearance, thin skin, and juicy, crispy and tasty flesh. When cultivated in open-field, DZ fruit production is greatly threatened by fruit cracking caused by rain (Ozturk et al., 2018). As facility cultivation can effectively control jujube fruit cracking, facility cultivated jujube are more and more popular in recent years. Moreover, facility warming practices revealed that a maximum temperature of 37 °C can greatly promote the ripening, advance their market entry time and raise the price of DZ fruits, and finally increase the farmers' income. In some facility cultivated jujube orchards, a maximum temperature of 42 °C was even used. Although this HT will lead to curled leaves and sometimes weakened tree and even whole tree death, 42 °C facility warming can result in more accelerated fruit ripening, better fruit coloration and higher price due to their earlier market entry and better appearance quality. Up to now, however, the influences of facility warming with different maximum temperatures on the development and quality of DZ fruits were not systematically investigated. In the present study, to explore the influences of facility warming on DZ fruits, fruit development- and quality-related parameters of DZ trees grown at three different maximum facility warming temperature (37 °C, 39.5 °C and 42 °C) at five stages (60 days post flowering (dpf), 70 dpf, 80 dpf, 90 dpf and 100 dpf) were studied.

*Piriformospora indica* (also named as *Serendipita indica*), an arbuscular mycorrhizal fungus (AMF)-like fungus that can form mutualistic symbioses with a wide range of plants (Mensah et al., 2020; Verma et al., 1998), has the ability of enhancing high temperature tolerance of host plants (Cheng et al., 2022). Studies also revealed that the *P. indica* culture filtrate (CF) also has similar promoting effects like the fungal colonization (Vahabi et al., 2015). To explore the possible application potential of this fungus in jujube industry, we also investigated the influences of CF spraying on DZ fruit development and ripening under facility warming condition. The results obtained in this study will provide insights into the mechanism of facility warming improved jujube fruit development and fruit quality, and can provide basis for the applications of CF in jujube cultivation.

## 2 Materials and methods

### 2.1 Materials and treatments

The *P. indica* strain (DSM11827) used in this study was provided by professor KaiWun-Yeh of Taiwan University and kept in our lab. Three *P. indica* fungal plugs (5 mm in diameter) were added to per 100 mL potato dextrose broth (PDB), shake-cultured at 200 rpm in dark at 28 °C for three days (Cheng et al., 2020), and filtrated using 8 layers of sterile cheesecloth. Then, filtrate was sterilized at 121 °C at 100 kPa for 20 min and diluted five times with distilled water to obtain CF for further use.

The ten-year-old DZ plants used in this study were cultivated in three adjacent simple temperature-controlled solar greenhouses located at Xunxia Family Farm (35°05′37.16″N, 110°52′55.76″E) in Linyi County, Yuncheng City, China. At 40 days post flowering (dpf), selected healthy DZ plants in each greenhouse were divided into two groups. Plants of one group were sprayed with 25 L CF using a manual sprayer every 10 days, for a total of three times (PI group), and plants of the other group were treated with equally diluted PDB and used as controls (CK group), At 60 dpf (late fruit expansion stage), fruit samples from the CK and PI groups (CK-T0 and PI-T0) without facility warming treatment were collected. Then, maximum temperature (when the temperature was higher than the set temperature, ventilation system will turn on automatically for cooling) of the three greenhouses was set to 37 °C (CK-T1 and PI-T1), 39.5 °C (CK-T2 and PI-T2), and 42 °C (CK-T3 and PI-T3), respectively. At 70 dpf (white ripen stage), 80 dpf (color breaking stage), 90 dpf (brittle ripen stage, suitable for harvesting), and 100 dpf (late brittle ripen stage), fruit samples were also collected. At least 100 fruits were collected from each group at each stage.

### 2.2 External fruit quality measurement

Fruit color parameters (L*, a*, and b* values) were measured on the equatorial surface of ten fruits using a CR8 colorimeter (3nh, Shenzhen, China). Fruit firmness (FF) was measured at the fruit equatorial region using a cylindrical probe (dimeter = 5 mm) on a texture analyzer (TMS-PRO, FTC, USA), with insertion speed of 1 mm/s, a probe travel distance of 10 mm, and an insertion depth of 20 mm. The longitudinal and transverse diameters (LD and TD) of DZ fruits were measured using a vernier caliper. The single fruit weight (SFW) was measured using an HZT-A + 100 electronic analytical balance (Hangzhou Baichuan Instrument Co., Ltd., Hangzhou, China). For the measurement of each parameter, ten DZ fruits were used for each group at each stage.

### 2.3 Determination of carotenoids, chlorophyll, anthocyanins and abscisic acid (ABA) contents in fruit peels

After grounding DZ fruit peels into fine powders in liquid nitrogen, 1 g DZ fruit peel powders were added into 5 mL acetone containing 0.1 % butylated hydroxytoluene (BHT), ultrasonic extracted for 1 h, and concentrated at 10000 rpm for 15 min to collect the supernatant. Then, absorbance values of collected supernatant at 663 nm, 645 nm and 450 nm were measured on a visible spectrophotometer (UV-1800, Shanghai Meixi Instrument Co., Ltd.), which were used for the calculations of the contents of chlorophyll *a*, chlorophyll *b*, total chlorophyll *a*nd carotenoids according to Yang, Yang, et al. (2023). For the determination of anthocyanins contents, 2.5 g fruit peel powders were added into extraction solution (containing 85 mL 95 % ethanol and 15 mL 1.5 M hydrochloric acid per 100 mL) to a final volume of 25 mL and kept in dark for 24 h. After measuring the optical density detection at 535 nm on a visible spectrophotometer (UV-1800, Shanghai Meixi Instrument Co., Ltd.), the anthocyanins content was calculated using the formula described by Yang, Yang, et al. (2023). ABA functions greatly in regulating the fruit ripening and fruit transition. In this study, the ABA content in fruit peel was also determined using a plant abscisic acid enzyme-linked immunosorbent assay kit (Jiankang Biological, Shanghai, China) according to the manual. At least three replications were made for the determinations of these parameters.

### 2.4 Physicochemical indices measurements

After removing kernels, DZ fruits were cut into small pieces, ground in a mortar and filtered through gauze to remove residues. Then, fruit filtrate was used for determinations of total soluble solids (TSS), soluble sugars (SS), titratable acid (TA), vitamin C (VC) and soluble protein (SP) content. TSS was measured using a WZB digital refractometer (Shanghai Yidi Physical Optics Instruments Co., Ltd., Shanghai, China). For the determinations of content of SS, TA, VC and SP, the anthrone colorimetric method (Jiang et al., 2020), the sodium hydroxide titration method (Jiang et al., 2020), the 2,6-dichloroindophenol colorimetric method (Yang, Yang, et al., 2023) and the Coomassie Brilliant Blue staining method was used, respectively. The starch content (SC) and sucrose content (SUC) were measured using starch and sucrose content assay kits produced by Beijing Solarbio Science & Technology Co., Ltd. (Beijing, China).

For the determinations of total phenols content (TPC) and total flavonoids content (TFC), kernel removed DZ fruits were first cut into small pieces, quickly frozen in liquid nitrogen and ground into a powder in a mortar. Two grams of fruit power was dissolved in 10 mL 80 % ethanol and extracted using an ultrasonic cleaner (2000TL, PRIMA, UK) for 15 min. Then, mixture was then centrifuged at 4 °C and 5000 rpm for 10 min. The supernatant was collected into a 25 mL brown volumetric flask. The extraction step was repeated once, and the final volume of supernatant was adjusted to 20 mL with 80 % ethanol and was used for determinations of TPC and TFC, and antioxidant capacities (2,2-diphenyl-1-picrylhydrazyl (DPPH) and 2,2′-azino-bis (3-ethylbenzothiazoline-6-sulfonicacid) (ABTS^+^) scavenging ability, and ferric-reducing antioxidant power (FRAP)). The TFC and ABTS^+^ of DZ fruits were measured according to the method of Fu et al. (2020). The TPC, DPPH and FRAP of DZ fruits were determined according to the method of Clarke et al. (2013).

### 2.5 Gene expression analysis

To investigate the influences of facility warming and CF treatments on DZ fruits from molecular levels, the expression patterns of carotenoids degradation related *9-cis-epoxycarotenoid dioxygenase* (*NCED*), starch degradation related *β-amylase* (*BAM*), sucrose biosynthesis related *sucrose-phosphate synthase* (*SPS*), flavonoids biosynthesis related *phenylalanine ammonia lyase* (*PAL*) and *chalcone synthase* (*CHS*), and anthocyanin biosynthesis related *dihydroflavonol-4- reductase* (*DFR*) gene in fruits of all treatment groups were studied using quantitative real time PCR (qRT-PCR). Encoded proteins of these selected genes were identified through BLASTP searches using TBtools (Yang, Yang, et al., 2023) with functionally verified *Arabidopsis* NCED, BAM, SPS, PAL, CHS and DRF as query sequences (Supplemental Table S1). Quantitative primers were designed based on their coding sequences using Primer 3.0 (Supplemental Table S2).

Total RNA was extracted from DZ fruits using a Trizol RNA Extraction Kit (TaKaRa, Dalian, China), and reverse transcribed into cDNA using a PrimeScript™ RT reagent Kit with gDNA Eraser (Perfect Real Time) kit (TaKaRa, Dalian, China). Amplifications were performed on a QuantStudio 3 (Applied Biosystems, Shanghai, China) quantitative real-time fluorescent PCR instrument using TB Green® Premix Ex Taq™ II kit (TaKaRa, Dalian, China) with three replications. By using *Actin* as the internal reference gene (Bu et al., 2016), the relative expression levels of these genes in DZ fruits were calculated using the 2^−∆∆Ct^ method.

### 2.6 Statical analysis

All data obtained in this study are expressed as mean ± standard deviation of at least three replicates. OriginPro 9.0 (OriginLab, Northampton, MA, USA) was used for the Pearson correlation analysis of physicochemical indices in DZ fruit peels and fruits, and for figure drawing. Principal component analysis (PCA) and redundancy analysis (RDA) of these indices were conducted using Canoco 5.0 software (Microcomputer Power, Ithaca, NY, USA). The significance of differences among DS samples was analyzed at *p* < 0.05 and/or *p* < 0.01 levels using one-way analysis of variance (ANOVA) in SPSS 25.0 (SPSS Inc., Chicago, IL, USA).

## 3 Results

### 3.1 Influences of facility warming and CF treatments on DZ fruit development

**Fig. 1:**
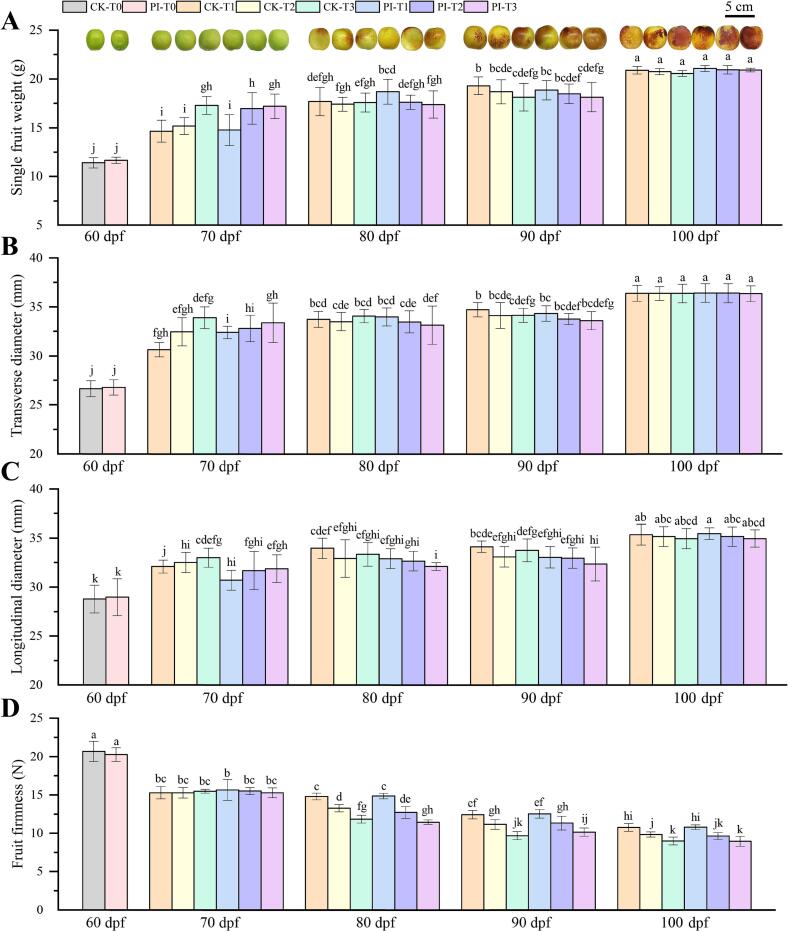
Single fruit weight (A), transverse diameter (B), longitudinal diameter (C) and fruit firmness (D) of DZ fruits from different groups at 60–100 days post flowering (dpf). CK and PI represents non-treated control group and *P. indica* culture filtrate treated group, respectively. T0: without facility warming; T1 ∼ T3: maximum temperature of 37 °C, 39.5 °C and 42 °C, respectively. Different letters above columns represent significant difference at *p* < 0.05 level.

As the fruits matured, the SFW, TD and LD of DZ fruits in each group showed an increasing trend (Fig. 1A-C), while the FF exhibited a decreasing trend (Fig. 1D). At 70 dpf, the higher the maximum temperature, the larger the values of SFW, TD and LD. The SFW of CK-T3 was significantly higher than that of CK-T2 and CK-T1 (*p* < 0.05), accounting for 1.14- and 1.18-fold of them, respectively. The SFW of PI-T2 and PI-T3 was significantly higher than that of PI-T1 (*p* < 0.05), accounting for 1.15- and 1.17-fold of it, respectively. Additionally, SFW of PI-T2 was about 1.12-fold of CK-T2 (*p* < 0.05), indicating that the CF treatment improved DZ fruit expansion at 39.5 °C at 70 dpf. Since 80 dpf, however, the SFW, TD and LD of fruits form DZ trees grown at 39.5 °C and 42 °C maximum temperature were smaller than that at 37 °C. This suggested that long-term facility warming suppressed the enlargement and development of DZ fruits. At 80 dpf, SFW of PI-T1 was significantly higher than that of PI-T3 (*p* < 0.05). At 90 dpf, SFW and TD of CK-T1 were both significantly higher than that of CK-T3 (*p* < 0.05). At 100 dpf, although no significant difference was identified, the average SFW of CF treated DZ fruits were larger than their corresponding controls.

### 3.2 Influences of facility warming and CF treatments on fruit color transition and pigmentation in DZ peels

By measuring the color parameters of DZ fruits, we found that the L* and b* values decreased while a* increased as fruit ripening (Table 1). Moreover, it was found that, as the facility warming temperature increased, the L* and b* values decreased while a* increased. At 70 dpf, the a* value of CK-T3 was significantly higher than that of CK-T1 and CK-T2 (*p* < 0.05), and the a* value of PI-T3 was also significantly higher than that of PI-T1 and PI-T2 (*p* < 0.05). These indicated that HT promoted the color transition of DZ fruits. Additionally, at 70 dpf, the a* value of PI-T1 and PI-T2 was significantly higher than that of CK-T1 and CK-T2, respectively (*p* < 0.05). Since 80 dpf, the a* values of CF treated DZ fruits were generally higher than their non-treated controls. These indicated that CF treatment further improved fruit coloration.

**Table 1:** Color parameters (L*, a* and b* values) and contents of anthocyanins, ABA, carotenoids, chlorophyll *a*, chlorophyll *b* and total chlorophylls in winter jujube fruit peels at different stages. CK and PI represents non-treated control group and *P. indica* culture filtrate treated group, respectively. T0: without facility warming; T1 ∼ T3: maximum temperature of 37 °C, 39.5 °C and 42 °C, respectively. Different letters (a–q) in each line indicate significant differences at the *p* < 0.05 level; FW: fresh weight; ABA: abscisic acid; Chl a: chlorophyll *a*; Chl b: chlorophyll *b*; Chl: total chlorophyll.

**Time**	**Group**	L*	a*	b*	**Anthocyanins** **(OD·mL/100 g FW)**	**ABA** **(ng/g)**	**Carotenoids** **(mg/kg)**	**Chl a** **(mg/kg)**	**Chl b** **(mg/kg)**	**Chl** **(mg/kg)**
60 dpf	CK-T0	62.69 ± 0.66^a^	−7.16 ± 0.56^n^	35.33 ± 0.76^a^	1.45 ± 0.02^lm^	648.48 ± 39.29^k^	14.39 ± 0.39^c^	28.48 ± 0.85^d^	9.65 ± 0.23^gh^	38.13 ± 1.08^h^
	PI-T0	62.75 ± 1.32^a^	−7.22 ± 0.38^n^	35.37 ± 0.46^a^	1.45 ± 0.01^lm^	648.4 ± 19.49^k^	14.56 ± 0.1^c^	23.94 ± 0.34^f^	9.35 ± 0.1^gh^	33.29 ± 0.41^i^
70 dpf	CK-T1	61.41 ± 1.25^b^	−6.53 ± 0.6^m^	31.76 ± 0.14^b^	1.12 ± 0.02^o^	715.62 ± 48.93^jk^	11.72 ± 0.16^e^	20.18 ± 0.81^h^	8.40 ± 0.61^ijk^	28.57 ± 0.85^k^
	CK-T2	61.26 ± 0.47^b^	−6.17 ± 0.54^m^	31.8 ± 0.31^b^	1.26 ± 0.01^n^	805.64 ± 40.04^hij^	11.60 ± 0.29^ef^	28.52 ± 0.12^d^	10.98 ± 0.4^ef^	39.5 ± 0.42^h^
	CK-T3	61.59 ± 0.3^b^	−4.17 ± 0.48^k^	31.57 ± 0.34^b^	1.5 ± 0.02^l^	852.92 ± 67.18^ghi^	18.60 ± 0.12^a^	37.70 ± 0.23^a^	32.71 ± 0.42^a^	70.41 ± 0.64^a^
	PI-T1	61.03 ± 0.55^b^	−5.56 ± 0.38^l^	31.71 ± 0.45^b^	1.37 ± 0.03^m^	771.95 ± 29.42^ij^	9.47 ± 0.82^i^	18.76 ± 1.08^i^	7.30 ± 2.04^klmn^	26.06 ± 3.12^l^
	PI-T2	61.58 ± 0.61^b^	−5.13 ± 0.43^l^	31.87 ± 0.86^b^	1.48 ± 0.01^l^	832.69 ± 35.26^ghi^	13.2 ± 0.11^d^	26.76 ± 0.14^e^	11.72 ± 0.17^e^	38.49 ± 0.28^h^
	PI-T3	61.49 ± 0.32^b^	−4.53 ± 0.6^k^	31.63 ± 0.34^b^	1.77 ± 0.01^ij^	857.79 ± 54.38^fghi^	16.08 ± 0.16^b^	32.73 ± 0.31^c^	27.53 ± 0.57^b^	60.27 ± 0.86^c^
80 dpf	CK-T1	55.78 ± 0.55^c^	−4.18 ± 0.34^k^	30.27 ± 0.05^c^	1.78 ± 0.01^ij^	853.58 ± 59.31^ghi^	15.63 ± 0.42^b^	33.32 ± 0.92^c^	28.13 ± 1.66^b^	61.45 ± 2.57^bc^
	CK-T2	54.79 ± 0.49^c^	−3.59 ± 0.41^j^	30.11 ± 0.29^c^	1.65 ± 0.09^k^	839.77 ± 53.67^ghi^	11.18 ± 0.30^f^	21.69 ± 0.69^g^	24.26 ± 0.85^c^	45.95 ± 1.53^f^
	CK-T3	54.58 ± 1.21^c^	−3.25 ± 0.58^j^	30.09 ± 0.39^c^	1.7 ± 0.02^jk^	884.53 ± 38.55^efgh^	8.15 ± 0.12^k^	15.76 ± 0.26^k^	6.61 ± 0.44^mn^	22.37 ± 0.63^mn^
	PI-T1	55.17 ± 1.33^c^	−3.62 ± 0.33^j^	30.4 ± 0.32^c^	1.79 ± 0.05^i^	868.8 ± 78.94^efghi^	15.96 ± 0.32^b^	27.98 ± 1.12^b^	28.14 ± 1.18^b^	62.57 ± 1.93^b^
	PI-T2	54.9 ± 0.23^c^	−3.27 ± 0.41^j^	30.15 ± 0.31^c^	1.68 ± 0.01^k^	859.57 ± 33.27^efghi^	14.18 ± 0.6^c^	34.43 ± 0.78^d^	27.63 ± 1.21^b^	55.61 ± 2.24^d^
	PI-T3	54.42 ± 0.25^c^	−2.46 ± 0.33^i^	30.33 ± 0.3^c^	1.88 ± 0.09^h^	921.49 ± 56.79^cdefg^	8.78 ± 0.07^j^	15.77 ± 0.15^k^	7.86 ± 0.14^jkl^	23.63 ± 0.29^m^
90 dpf	CK-T1	46.37 ± 0.85^d^	−1.03 ± 0.31^h^	28.42 ± 1.43^d^	1.94 ± 0.00^gh^	870.11 ± 58.25^efgh^	7.15 ± 0.16^l^	12.07 ± 0.20^m^	6.91 ± 0.35^lmn^	18.98 ± 0.46^o^
	CK-T2	44.63 ± 1.55^e^	0.26 ± 0.04^f^	27.95 ± 0.25^de^	1.96 ± 0.02^fgh^	893.93 ± 66.1^defgh^	9.65 ± 0.32^hi^	18.27 ± 0.78^ij^	9.92 ± 1.21^fg^	28.19 ± 1.99^k^
	CK-T3	41.34 ± 1.34^f^	1.98 ± 0.13^e^	26.60 ± 0.31^f^	2.19 ± 0.04^e^	956.17 ± 57.35^bcde^	10.23 ± 0.51^g^	15.43 ± 0.74^k^	10.3 ± 1.48^fg^	25.73 ± 2.22^l^
	PI-T1	46.29 ± 1.03^d^	−0.25 ± 0.04^g^	28.23 ± 0.87^d^	1.94 ± 0.00^gh^	872.23 ± 41.53^efgh^	9.81 ± 0.10^ghi^	17.64 ± 0.26^j^	8.64 ± 0.08^hij^	26.28 ± 0.32^l^
	PI-T2	44.21 ± 0.09^e^	0.48 ± 0.03^f^	27.31 ± 1.03^e^	2.18 ± 0.07^e^	915.5 ± 78.89^cdefg^	10.04 ± 0.38^gh^	18.58 ± 0.58^i^	12.05 ± 1.16^e^	30.63 ± 1.73^j^
	PI-T3	41.05 ± 0.79^f^	1.99 ± 0.22^e^	26.52 ± 0.99^f^	2.57 ± 0.08^b^	986.27 ± 78.78^abcd^	12.80 ± 0.31^d^	21.67 ± 0.49^g^	21.5 ± 0.82^d^	43.17 ± 1.31^g^
100 dpf	CK-T1	39.03 ± 0.7^g^	2.92 ± 0.30^d^	24.95 ± 1.33^g^	1.95 ± 0.01^gh^	922.87 ± 35.15^cdefg^	11.71 ± 0.4^e^	21.99 ± 0.58^g^	24.39 ± 0.98^c^	46.38 ± 1.55^f^
	CK-T2	38.92 ± 0.82^g^	3.59 ± 0.31^c^	23.64 ± 1.55^h^	2.04 ± 0.04^f^	952.11 ± 113.63^cdef^	6.2 ± 0.18^m^	11.78 ± 0.07^m^	7.60 ± 0.34^jklm^	19.38 ± 0.4^o^
	CK-T3	36.86 ± 1.07^h^	4.25 ± 0.34^b^	22.88 ± 1.34^i^	2.42 ± 0.06^c^	1050.86 ± 82.04^ab^	5.37 ± 0.05^n^	8.81 ± 0.21^n^	6.22 ± 0.38^n^	15.03 ± 0.58^p^
	PI-T1	38.89 ± 1.15^g^	3.56 ± 0.34^c^	24.44 ± 1.89^g^	1.99 ± 0.01^gh^	927.51 ± 80^cdefg^	15.77 ± 0.85^b^	19.77 ± 2.08^h^	31.98 ± 0.09^a^	52.25 ± 0.53^e^
	PI-T2	38.32 ± 0.38^g^	3.87 ± 0.39^bc^	23.43 ± 0.68^hi^	2.29 ± 0.05^d^	992.43 ± 61.2^abc^	6.83 ± 0.18^l^	13.02 ± 0.33^l^	7.70 ± 0.49^jklm^	20.73 ± 0.81^no^
	PI-T3	36.19 ± 0.60^h^	5.06 ± 0.31^a^	22.80 ± 0.15^i^	2.70 ± 0.06^a^	1055.23 ± 77.19^a^	5.95 ± 0.05^m^	7.56 ± 0.42^o^	2.84 ± 0.87^o^	10.41 ± 1.29^q^

The change patterns of anthocyanins and ABA contents were almost the same to that of the a* value (Table 1). At 70 dpf, the anthocyanins contents of CK-T2 and CK-T3 were both significantly higher than that of CK-T1 (*p* < 0.05); the anthocyanins content of PI-T3 was significantly higher than that of PI-T1 and PI-T2 (*p* < 0.05). Additionally, under the same temperature, the anthocyanins contents in CF treated DZ peels were higher than their non-treated controls at different stages (*p* < 0.05), indicating that CF application promoted the anthocyanins accumulation in DZ fruit peel. The contents of carotenoids and chlorophylls in DZ fruit peels were also determined (Table 1). Since 70 dpf, the contents of carotenoids, chlorophyll a, chlorophyll *b*, and total chlorophyll in DZ peel of all treatment groups displayed a ‘rise-fall-rise’ trend.

**Fig. 2:**
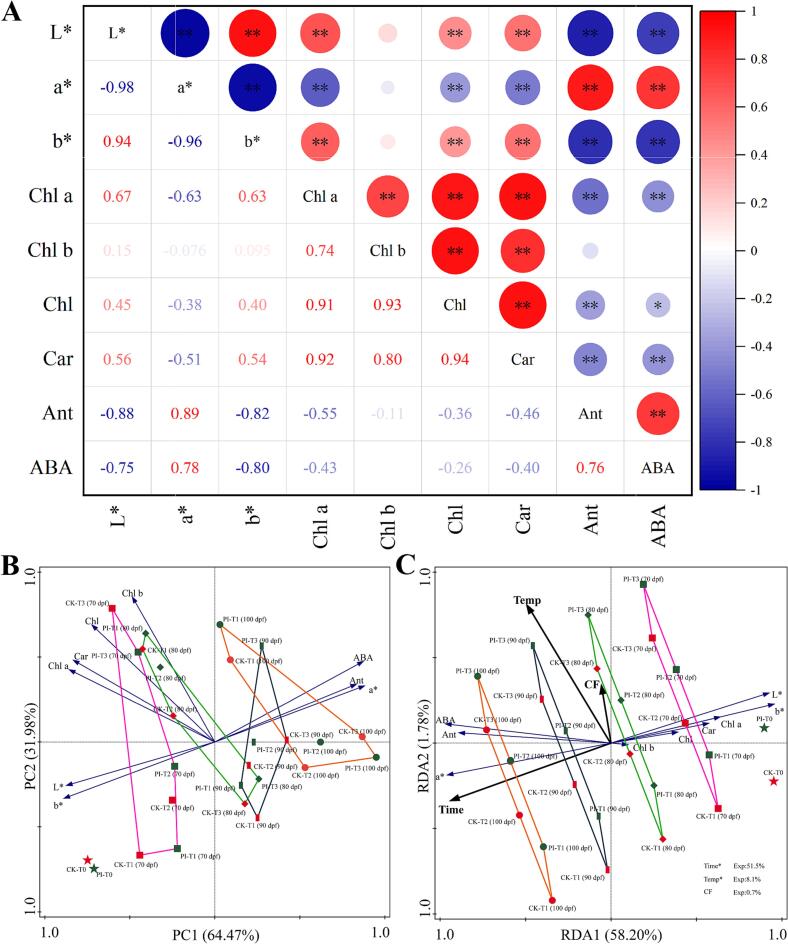
Correlation analysis (A), PCA (B), and RDA (C) results for parameters of winter jujube fruit peels. CK and PI represents non-treated control group and *P. indica* culture filtrate treated group, respectively. T0: without facility warming; T1 ∼ T3: maximum temperature of 37 °C, 39.5 °C and 42 °C, respectively. * and ** indicates significant correlation (*p* < 0.05) and very significant correlation (*p* < 0.01), respectively. Chl: total chlorophyll; Chl a: chlorophyll *a*; Chl b: chlorophyll *b*; Car: carotenoids; ABA: abscisic acid; Ant: anthocyanins.

To further study the influences of facility warming and CF on the color transition and pigments accumulation, we conducted correlation analysis, principal component analysis (PCA) and redundancy analysis (RDA) of DZ fruit peel parameters (Fig. 2). Correlation analysis results revealed very significant positively correlations among the a* value, anthocyanins content and ABA content, and these parameters were all significantly or very significantly negatively correlated with L* and b* values, and chlorophyll *a*, total chlorophyll *a*nd carotenoids contents (Fig. 2A). In consistence with the correlation analysis results, our PCA results also revealed that a*, anthocyanins content, and ABA content in DZ fruit peel are positively correlated with each other, and negatively correlated with b*, L*, and contents of chlorophyll *a*, total chlorophyll, and carotenoids (Fig. 2B). The a* value has the highest positive contribution to PC1, and the a* value, anthocyanins content, and ABA content of PI-T3 (at 100 dpf) scored the highest on PC1. Classification based on the five different stages revealed that, with the extension of facility warming treatment, anthocyanins content, a*, and ABA content in DZ fruit peel increased, while L* and b* values decreased (Fig. 2B). Furthermore, our RDA results revealed that facility warming treatment time length (time) is the most significant factor affecting these peel parameters (explaining 51.5 % of the variations, *p* < 0.05), followed by temperature (explaining 8.1 % of the variations, *p* < 0.05) (Fig. 2C). Time and temperature are positively correlated with each other and positively correlated with a*, anthocyanins and ABA contents, but negatively correlated with L*, b*, and contents of carotenoids, chlorophyll a, chlorophyll *b*, and total chlorophyll. Moreover, time is negatively correlated with CF, while temperature is positively correlated with CF. It should be noted that CF is also positively correlated with anthocyanins and ABA contents in DZ fruit peel.

### 3.3 Influences of facility warming and CF treatments on fruit internal quality and antioxidant capacities

As the fruit ripened, the SS, TSS, SUC, SC, TA, and SP contents in DZ fruits from all groups increased, while the VC content decreased (Table 2). At 80 dpf, the VC content of CF treated fruits were all significantly higher than that of their non-treated controls (*p* < 0.05). At 100 dpf, the SS, TSS, SUC, SC, TA, and SP contents in PI-T3 were all the highest. The VC content of CK-T1 and PI-T1 was significantly higher than that of CK-T3 and PI-T3 (*p* < 0.05), respectively.

**Table 2:** Fruit internal quality parameters of winter jujube fruits at different stages. Different letters (a–o) in each line indicate significant differences at the *p* < 0.05 level; CK and PI represents non-treated control group and *P. indica* culture filtrate treated group, respectively. T0: without facility warming; T1 ∼ T3: maximum temperature of 37 °C, 39.5 °C and 42 °C, respectively. SS: soluble sugars; TSS: total soluble solids; SC: starch content; SUC: sucrose content; TA: titratable acid; VC: vitamin C; SP: soluble protein.

**Time**	**Group**	**SS (%)**	**TSS (%)**	**SUC (mg/g)**	**SC (mg/g)**	**TA (%)**	**SP (mg/g)**	**VC (mg/100 g)**
60 dpf	CK-T0	9.22 ± 0.26^m^	10.70 ± 0.37^l^	28.55 ± 0.88^n^	17.19 ± 0.41^n^	0.18 ± 0.03^i^	6.64 ± 0.36^ghi^	350.25 ± 3.68^a^
	PI-T0	9.24 ± 0.16^m^	10.72 ± 0.55^l^	28.69 ± 1.51^n^	17.19 ± 0.35^n^	0.17 ± 0.03^i^	6.62 ± 0.55^ghi^	350.28 ± 1.39^a^
70 dpf	CK-T1	10.16 ± 0.16^lm^	11.78 ± 0.33^k^	30.37 ± 1.59^nm^	19.20 ± 1.20^m^	0.18 ± 0.03^i^	5.44 ± 0.48^j^	324.01 ± 7.47^b^
	CK-T2	10.49 ± 0.33^kl^	11.76 ± 0.32^k^	33.57 ± 1.46^lm^	20.07 ± 0.99^lm^	0.19 ± 0.00^hi^	6.43 ± 0.15^hi^	305.95 ± 6.72^cd^
	CK-T3	11.35 ± 0.44^jk^	11.54 ± 0.23^k^	38.23 ± 1.61^k^	21.5 ± 0.29^jkl^	0.19 ± 0.02^hi^	6.55 ± 0.69^ghi^	301.51 ± 1.53^de^
	PI-T1	10.43 ± 0.22^kl^	11.84 ± 0.11^k^	31.96 ± 1.12^lmn^	19.52 ± 0.87^m^	0.19 ± 0.03^hi^	6.15 ± 0.49^ij^	325.23 ± 2.17^b^
	PI-T2	11.14 ± 0.37^jk^	11.92 ± 0.6^k^	36.45 ± 3.06^kl^	20.87 ± 0.43^klm^	0.20 ± 0.01^hi^	6.62 ± 0.44^ghi^	311.54 ± 1.71^c^
	PI-T3	11.63 ± 0.16^j^	11.96 ± 0.36^k^	38.26 ± 3.89^k^	22.4 ± 1.06^ijk^	0.21 ± 0.01^fghi^	6.73 ± 0.38^ghi^	303.44 ± 2.05^de^
80 dpf	CK-T1	13.45 ± 0.57^i^	14.40 ± 0.16^j^	39.39 ± 1.63^jk^	22.19 ± 1.52^ijk^	0.19 ± 0.01^hi^	6.18 ± 0.97^ij^	302.15 ± 5.19^de^
	CK-T2	13.85 ± 0.17^i^	14.50 ± 0.16^j^	43.79 ± 2.58^j^	22.66 ± 0.27^ij^	0.19 ± 0.00^hi^	7.18 ± 0.6^efgh^	284.35 ± 3.86^ghi^
	CK-T3	14.32 ± 0.60^hi^	14.84 ± 0.19^ij^	58.01 ± 5.92^i^	23.13 ± 0.69^ij^	0.20 ± 0.02^ghi^	7.49 ± 0.41^cdefg^	277.08 ± 11.42^ijk^
	PI-T1	13.80 ± 0.65^i^	14.74 ± 0.36^j^	39.39 ± 1.11^jk^	22.97 ± 0.59^ij^	0.18 ± 0.00^hi^	6.20 ± 0.24^ij^	321.35 ± 4.50^b^
	PI-T2	13.96 ± 0.56^hi^	14.92 ± 0.43^ij^	54.45 ± 2.78^i^	23.82 ± 0.59^i^	0.23 ± 0.02^fgh^	7.40 ± 0.69^defg^	296.19 ± 3.59^ef^
	PI-T3	14.84 ± 0.13^gh^	15.30 ± 0.24^hi^	62.96 ± 1.71^h^	23.62 ± 0.38^i^	0.27 ± 0.02^bcde^	7.75 ± 0.31^bcdef^	285.38 ± 3.86^gh^
90 dpf	CK-T1	15.43 ± 0.16^fg^	15.56 ± 0.17^gh^	63.87 ± 2.28^gh^	26.22 ± 1.81^h^	0.23 ± 0.01^efgh^	7.09 ± 0.4^fghi^	287.43 ± 2.73^gh^
	CK-T2	15.63 ± 0.40^efg^	16.02 ± 0.39^fg^	73.51 ± 3.94^f^	28.87 ± 1.64^g^	0.25 ± 0.01^cdef^	7.40 ± 0.38^defg^	280.62 ± 6.96^hij^
	CK-T3	16.46 ± 0.23^de^	17.36 ± 0.36^e^	85.23 ± 2.58^e^	32.96 ± 1.25^ef^	0.28 ± 0.02^bcd^	7.84 ± 1.09^bcdef^	264.97 ± 4.77^l^
	PI-T1	15.74 ± 0.57^efg^	15.62 ± 0.28^fgh^	67.77 ± 2.42^g^	26.63 ± 0.41^h^	0.19 ± 0.01^hi^	7.24 ± 0.67^efgh^	289.85 ± 4.24^fg^
	PI-T2	16.07 ± 0.67^def^	16.34 ± 0.11^f^	77.57 ± 2.24^f^	31.53 ± 0.47^f^	0.24 ± 0.03^defg^	7.68 ± 0.45^cdef^	284.91 ± 5.61^gh^
	PI-T3	16.81 ± 0.26^d^	17.74 ± 0.67^e^	86.17 ± 2.86^e^	33.52 ± 0.57^e^	0.29 ± 0.01^bc^	8.35 ± 1.06^abc^	276.24 ± 6.07^jk^
100 dpf	CK-T1	19.22 ± 0.13^c^	19.80 ± 0.16^d^	99.28 ± 4.54^d^	34.28 ± 1.4^de^	0.28 ± 0.02^bcd^	8.01 ± 0.82^abcdef^	263.66 ± 4.1l^m^
	CK-T2	20.13 ± 0.33^bc^	21.58 ± 0.16^b^	109.22 ± 2.42^c^	38.17 ± 1.83^c^	0.28 ± 0.02^bcd^	8.29 ± 0.5^abcd^	253.51 ± 4.21^n^
	CK-T3	21.43 ± 1.19^a^	23.26 ± 0.21^a^	113.89 ± 4.26^b^	41.35 ± 0.88^b^	0.31 ± 0.03^ab^	8.65 ± 0.59^ab^	239.99 ± 3.99^o^
	PI-T1	19.37 ± 0.31^bc^	19.92 ± 0.19^c^	102.15 ± 3.08^d^	35.32 ± 1.18^d^	0.28 ± 0.02^bcd^	8.08 ± 0.26^abcde^	271.12 ± 3.20^kl^
	PI-T2	20.22 ± 0.25^b^	21.44 ± 0.42^b^	113.09 ± 3.12^bc^	41.61 ± 0.80^b^	0.31 ± 0.04^ab^	8.65 ± 0.35^ab^	257.14 ± 4.67^mn^
	PI-T3	22.20 ± 1.05^a^	23.38 ± 0.47^a^	118.69 ± 4.97^a^	43.61 ± 1.78^a^	0.34 ± 0.04^a^	8.91 ± 0.00^a^	243.81 ± 2.62^o^

**Fig. 3:**
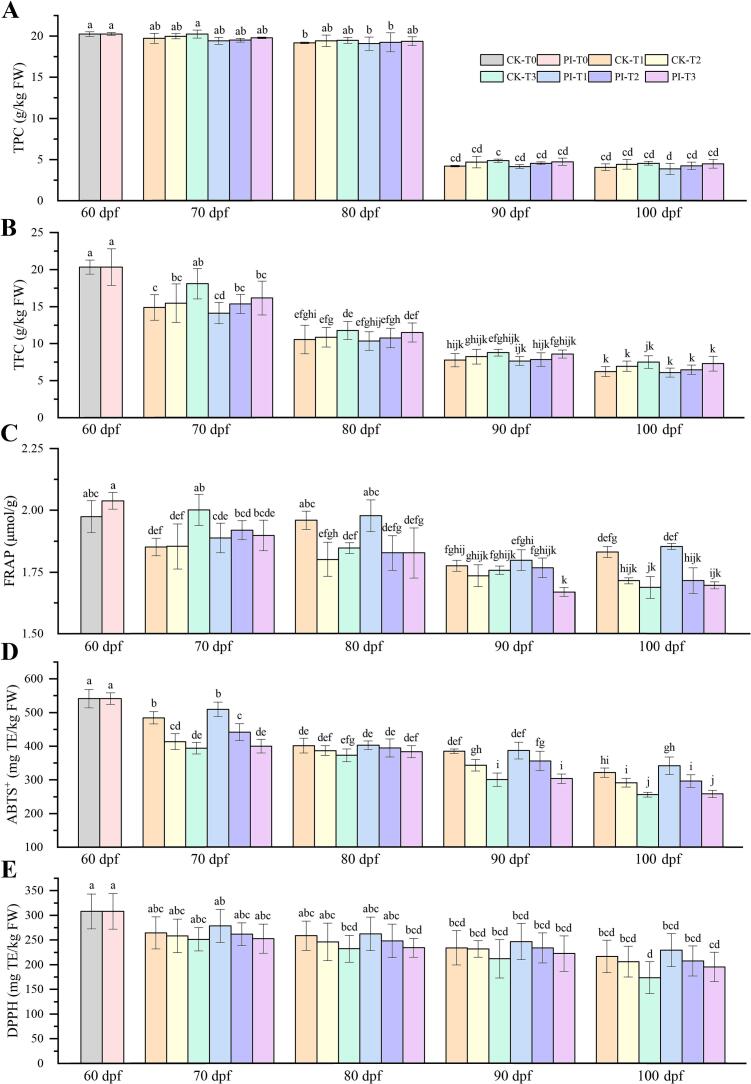
Contents of total phenols (A) and total flavonoids (B), and capacities of FRAP (C), ABTS^+^ (D) and f DPPH (E) in winter jujube fruits at 60–100 days post flowering (dpf). CK and PI represents non-treated control group and *P. indica* culture filtrate treated group, respectively. T0: without facility warming; T1 ∼ T3: maximum temperature of 37 °C, 39.5 °C and 42 °C, respectively. TE: Trolox equivalent; FW: fresh weight; TPC: total phenols content; TFC: total flavonoids content; DPPH: 2,2-diphenyl-1-picrylhydrazyl; ABTS^+^: 2,2′-azino-bis (3-ethylbenzothiazoline-6-sulfonicacid); FRAP: ferric-reducing antioxidant power.

As the fruit ripened, the TPC, TFC, FRAP, ABTS^+^, and DPPH in DZ fruits of all groups decreased (Fig. 3A-E). Higher maximum temperature led to increased TPC and TFC, and decreased FRAP, ABTS^+^, and DPPH in DZ fruits. Since 70 dpf, CF treated fruits were of relatively higher ABTS^+^ and DPPH capacities than their corresponding non-treated controls.

### 3.4 Influences of facility warming and CF treatments on the expression of fruit quality-related genes

**Fig. 4:**
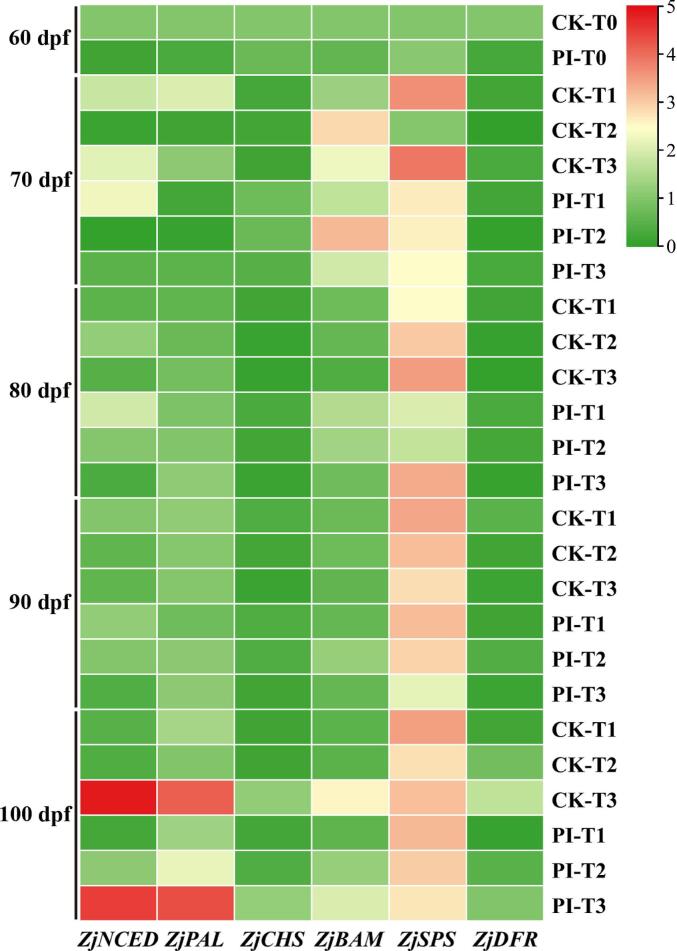
Heatmap for the expression patterns of carotenoids-, starch-, sucrose-, flavonoids-, and anthocyanin-related genes in facility warming and CF treated winter jujube fruits at five different stages. For the heatmap drawing, log_2_ (Relative expression level value +1) was used. CK and PI represents non-treated control group and *P. indica* culture filtrate treated group, respectively. T0: without facility warming; T1 ∼ T3: maximum temperature of 37 °C, 39.5 °C and 42 °C, respectively. *NCED*: *9-cis-epoxycarotenoid dioxygenase*; *BAM*: *β-amylase*; *SPS*: s*ucrose-phosphate synthase*; *PAL*: *phenylalanine ammonia lyase*; *CHS*: *chalcone synthase*; *DFR*: *dihydroflavonol-4- reductase*.

Quantitative real time PCR (qRT-PCR) was applied to validate the expression of *ZjNCED*, *ZjBAM*, *ZjSPS*, *ZjPAL*, *ZjCHS*, and *ZjDFR* genes in facility warming and CF treated DZ fruits (Fig. 4). At 90 dpf, higher temperature resulted in lower expression of *ZjNCED*, while CF treatment upregulated its expression. At 70–90 dpf, HT downregulated the expression of *ZjPAL*, while CF treatment moderately upregulated its expression. At 100 dpf, however, the higher the temperature, the higher the expression level of *ZjPAL*, and its expression in CF treated fruits was higher than the non-treated controls. The relative expression levels of *ZjCHS* in CF treated fruits were generally higher than their corresponding non-treated controls. Except for CK-T1 and CK-T3 at 70 dpf, the expression levels of *ZjSPS* in fruits from both CK and PI groups increased as fruit ripened, and its expression in CF treated fruits was lower than non-treated controls. At 100 dpf, the expression of *ZjBAM* was upregulated by HT, and its expression in CK-T3 was higher than in PI-T3.

### 3.5 Correlation analysis, PCA, and RDA of quality- and antioxidant capacity-related parameters in DZ fruits

**Fig. 5:**
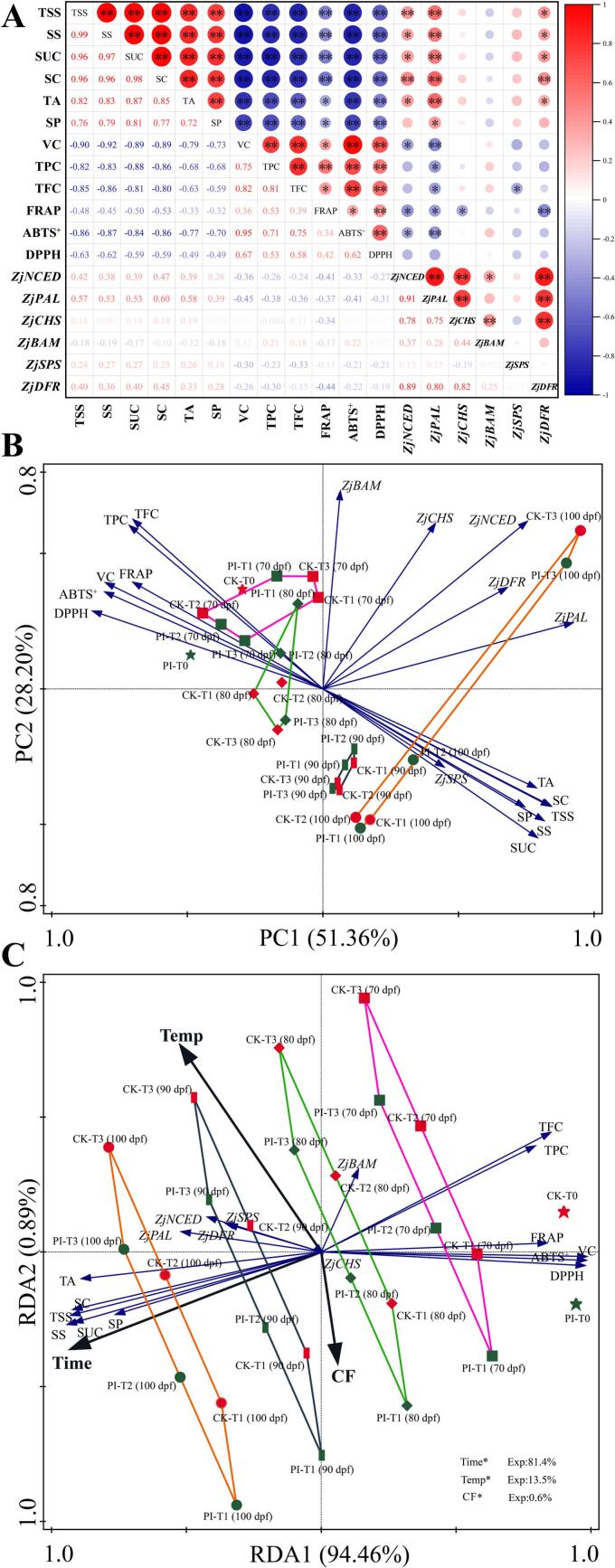
Correlation analysis (A), PCA (B), and RDA (C) of fruit quality- and fruit antioxidant activity-related parameters in winter jujube fruits. CK and PI represents non-treated control group and *P. indica* culture filtrate treated group, respectively. T0: without facility warming; T1 ∼ T3: maximum temperature of 37 °C, 39.5 °C and 42 °C, respectively. * and ** indicates significant correlation (*p* < 0.05) and very significant correlation (*p* < 0.01), respectively. SS: soluble sugars; TSS: total soluble solids; SC: starch content; SUC: sucrose content; TA: titratable acid; VC: vitamin C; SP: soluble protein; TPC: total phenols content; TFC: total flavonoids content; DPPH: 2,2-diphenyl-1-picrylhydrazyl; ABTS^+^: 2,2′-azino-bis (3-ethylbenzothiazoline-6-sulfonicacid); FRAP: ferric-reducing antioxidant power; *NCED*: *9-cis-epoxycarotenoid dioxygenase*; *BAM*: *β-amylase*; *SPS*: s*ucrose-phosphate synthase*; *PAL*: *phenylalanine ammonia lyase*; *CHS*: *chalcone synthase*; *DFR*: *dihydroflavonol-4- reductase*.

Correlation analysis revealed significant or very significant positive correlations among TSS, SS, SUC, SC, TA, and SP, among VC, TPC, TFC, FRAP, ABTS^+^, and DPPH, and among *ZjNCED*, *ZjPAL*, and *ZjCHS* expression levels (Fig. 5A). Interestingly, fruit internal quality related parameters (TSS, SS, SUC, SC, TA, and SP) were found to be significantly or very significantly negatively correlated with antioxidant activity-related parameters (VC, TPC, TFC, FRAP, ABTS^+^, and DPPH). The expression of *ZjBAM* is significantly positively correlated with *ZjNCED* and negatively correlated with SC. The expression of *ZjCHS* is positively correlated with TFC, and the expression of *ZjSPS* is positively correlated with SUC. Moreover, the expression of *ZjNCED*, *ZjPAL*, and *ZjCHS* were all significantly positively correlated with that of *ZjDFR*. These correlations were also well supported by our PCA and RDA results (Fig. 5B and C).

PCA results revealed that DPPH and SUC had the highest negative contribution rate to PC1 and PC2, while *ZjPAL* and *ZjBAM* had the highest positive contribution rate to PC1 and PC2 (Fig. 5B), respectively. Classification based on fruit stages revealed that, as the facility warming time prolonged, the SS, SP, SUC, SC, and TA contents in DZ fruits increased, while TFC, TPC, VC, and antioxidant capacities decreased.

RDA revealed that temperature, time, and CF significantly influenced the quality parameters of DZ fruits (*p* < 0.05) and were important environmental variables associated with fruit quality- and antioxidant activity-related parameters, explaining 81.4 %, 13.5 %, and 0.6 % of variations (Fig. 5C), respectively. Time was positively correlated with temperature and CF, while temperature was negatively correlated with CF. Time and temperature were positively correlated with *ZjSPS*, *ZjDFR*, *ZjNCED*, *ZjPAL*, TA, SC, TSS, SS, SUC, and SP, and negatively correlated with TFC, TPC, FRAP, VC, ABTS^+^, DPPH, and *ZjCHS*. Additionally, CF was found to be positively correlated with *ZjCHS*, DPPH, ABTS^+^, VC, SP, and SUC, and negatively correlated with *ZjBAM*, *ZjSPS*, TPC, and TFC.

## 4 Discussion

### 4.1 Facility warming accelerates DZ fruits ripening and short-term facility warming can promote fruit enlargement

Evidences revealed that moderate high temperature can promote the maturation of crops and fruits. In rice, when the temperature is raised by 2.0 °C and 4.0 °C, the growth period of rice was shortened by 6 d and 12 d (Sanwong et al., 2023), respectively; compared to rice grown at 22 °C, the grain filling period of rice grown at 32 °C was shortened by 26 d to 32 d (Ahmed et al., 2015); a 5 °C temperature increasement would lead to improved TD, LD, and SFW of grains of heat-tolerant rice (She et al., 2010). In grape, a 4 °C temperature increasement could shorten the transition period from mid-coloring to ripening by 12 d, accelerate development, and advance ripening of grapes (Arrizabalaga-Arriazu et al., 2020). In apple, it was also reported that HT can promote the growth and ripening of apple fruits (Hatfield & Prueger, 2015). In this study, we found that, at 70 dpf, the SFW, TD, and LD of DZ fruits increased as the maximum temperature increased. However, since 80 dpf, these parameters of the 39.5 °C and 42 °C group were smaller than that of the 37 °C group. At 100 dpf, the DZ fruit weight of the 42 °C group was the smallest. These indicated that short-term facility warming promoted fruit enlargement, while long-term facility warming reduced the fruit size and weight. In consistence with our results, similar findings were discovered in rice (Arshad et al., 2017).

### 4.2 Facility warming accelerates peel color transition and fruit quality formation, but decreases the antioxidant ability of DZ fruits

Anthocyanins are one of the main pigments responsible for the coloration of many fruits (Li, Hong, et al., 2021). Under HT, the anthocyanins accumulation in plum fruits (Niu et al., 2017) was enhanced. Consistently, our study showed that the anthocyanins content in DZ peels increased with rising temperature. ABA is a major phytohormone involved in fruit ripening regulation and is also an important player in plant stress responses (Fenn & Giovannoni, 2021). Under HT stress, the ABA content in tomato (Li et al., 2022) increased rapidly. Our study showed that facility warming promoted ABA accumulation in DZ fruits, and revealed a very significant positive correlation among ABA content, anthocyanins content, and a* value in DZ peels. These indicated that facility warming promoted the peel color transition and fruit ripening of DZ by enhancing ABA and anthocyanins biosynthesis.

HT can increase the VC contents in lettuce (Li et al., 2020). However, HT led to VC decreasing in tomato fruits (Massot et al., 2013). These indicated that the effects of HT on VC accumulation varied in different plant species. Our study found that the VC content in DZ fruits decreased as the maximum temperature increased. HT can increase the sugar content in grapes (Arrizabalaga-Arriazu et al., 2020). The TSS content in fruits of tomato grown at 36 °C is higher than that grown at 32.4 °C (Vijayakumar et al., 2021). Under HT, the SC of rice increased (Vijayakumar et al., 2021). Our study revealed a positive correlation between temperature and contents of SS, TSS, and SC in DZ fruits, i.e. the higher the temperature, the higher these fruit quality-related parameters. Moreover, our qRT-PCR analysis results revealed significant negative correlation between expression of *ZjBAM* and SC, and positive correlation between expression of *ZjSPS* and SUC. These results suggested that facility warming and CF treatments affected the expression of starch and sucrose metabolism related structural genes, and promoted the accumulation of SS, TSS, and SC in DZ fruits.

CHS is a key enzyme catalyzing the flavonoids biosynthesis (Bartas et al., 2023). Researches revealed that HT can increase plant flavonoids accumulation (Vijayakumar et al., 2021). Similarly, our study found that the higher the facility warming temperature, the higher the TPC and TFC and expression of *ZjCHS* in fruits. Additionally, the expression of *ZjCHS* was identified to be positively correlated with both TPC and TFC in DZ fruits. Thus, it was hypothesized that facility warming increased the phenols and flavonoids accumulation in DZ fruits, at least partially, by influencing the expression of *ZjCHS*.

### 4.3 CF treatment promotes anthocyanins accumulation in fruit peel, and enhance antioxidant capacity of DZ fruits

*P. indica* treatment can increase the anthocyanins content in black rice (Solanki et al., 2023). In tomato, *P. indica* can also increase the carotenoids and anthocyanins contents in tomato fruits under HT condition (Yan et al., 2021). In our study, the anthocyanins contents of CF treated DZ fruits were mostly higher and even significantly higher than their corresponding non-treated controls since 70 dpf, and their fruits had a deeper red color (higher a* value). This indicates that CF treatment can promote the anthocyanins accumulations in DZ fruit peel.

*P. indica* treatment can enhance plant antioxidant capacity and improve plant tolerance to abiotic stresses (Wang et al., 2023). Under stress conditions, *P. indica* colonization can activate the antioxidant defense mechanisms of host plants, inhibit the production of reactive oxygen species and alleviate the adverse effects of stress on plants (Liu et al., 2021). It was reported that *P. indica* can enhance Chinese cabbage's tolerance to HT (Hua et al., 2017), as well as improve the heat tolerance of bananas (Bodjrenou et al., 2022). Our study found that CF treated DZ fruits had relatively higher FRAP, DPPH, and ABTS^+^ capacities compared to non-treated fruits, indicating that CF application can enhance the antioxidant ability of DZ fruits.

## 5 Conclusion

**Fig. 6:**
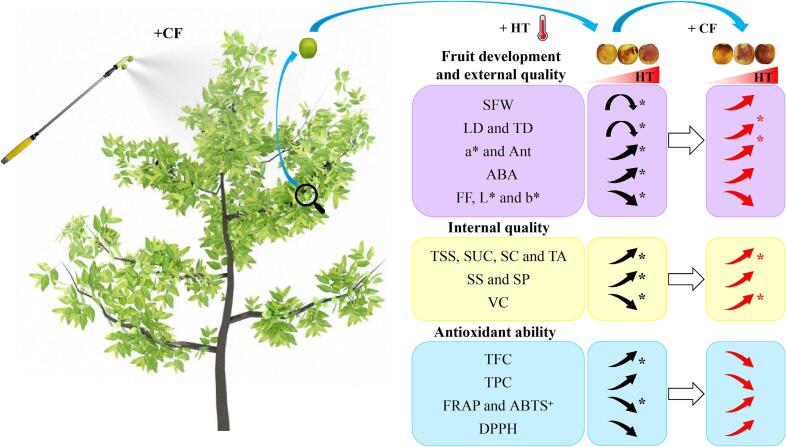
Effects of facility warming and *P. indica* culture filtrate treatments on winter jujube fruits. Turning arrow represents ‘rise-fall’ change pattern of fruit parameters. The arrow points represent change patterns of fruit parameters during fruit ripening. Black and red arrows represent the influence of different maximum temperature and CF on the fruit parameters, respectively. Black asterisks represent significant difference caused by different maximum temperature. Red asterisks represent significant difference caused by CF compared to non-treated controls. HT: high temperature; CF: *P. indica* culture filtrate; LD: longitudinal diameter; TD: transverse diameter; SFW: single fruit weight; Ant: anthocyanins; ABA: abscisic acid; FF: fruit firmness; SS: soluble sugars; TSS: total soluble solids; SC: starch content; SUC: sucrose content; TA: titratable acid; VC: vitamin C; SP: soluble protein; TPC: total phenols content; TFC: total flavonoids content; DPPH: 2,2-diphenyl-1-picrylhydrazyl; ABTS^+^: 2,2′-azino-bis (3-ethylbenzothiazoline-6-sulfonicacid); FRAP: ferric-reducing antioxidant power. (For interpretation of the references to color in this figure legend, the reader is referred to the web version of this article.)

In this study, we investigated the influences of facility warming at three different maximum temperatures on the development, coloration, quality formation and antioxidant ability of winter jujube fruits. Results revealed that short-term facility warming can accelerate fruit enlargement, but long-term HT would lead to reduced fruit size and weight. Facility warming can promote SS, TSS, TA, SP, SUC, and SC contents in DZ fruits, but would lead to decreased VC content and suppressed antioxidant ability. Notably, these effects are more significant as temperature rises. The CF treatment can further improve the positive effects of HT on DZ fruits. Moreover, CF treatment can increase the VC content and slightly improve the antioxidant ability of DZ fruits under high temperature (Fig. 6).

## Funding

The work supported by the Science and Technology Achievement Transformation Guiding Special Project of Shanxi Province (202204021301033), the Bio-breeding Engineering Project of Shanxi Agricultural University (YZGC110), the Pairing Assistance Cooperation Project of Shanxi Province (202104041101038), and the Open Competition Project of Shanxi Provincial Science and Technology Department (202201140601027).

## CRediT authorship contribution statement

**Anping Shao:** Writing – original draft, Formal analysis, Data curation. **Junqiang Yang:** Resources, Funding acquisition, Conceptualization. **Hao Li:** Visualization, Validation. **Ruide Li:** Validation, Data curation. **Yang Hu:** Validation, Data curation. **Chunzhen Cheng:** Writing – review & editing, Writing – original draft, Supervision, Project administration, Conceptualization.

## Declaration of competing interest

The authors declare that they have no known competing financial interests or personal relationships that could have appeared to influence the work reported in this paper.

## Data Availability

Data will be made available on request.

## Appendix A Supplementary data

Supplementary material 1 **Supplemental Table S1:** Information for the identified carotenoids degradation related *ZjNCED*, starch degradation related *ZjBAM*, sucrose biosynthesis related *ZjSPS*, flavonoids biosynthesis related *ZjPAL* and *ZjCHS*, and anthocyanin biosynthesis related *ZjDFR* in winter jujube fruits. Supplementary material 1

Supplementary material 2 **Supplemental Table S2:** Information for the primers used in this study. Supplementary material 2
