# Supplementary material for: Piriformospora indica culture filtrate application adds brilliance to the promoting effects of facility warming on winter jujube fruit ripening
Source: Food Chem X. 2024 Nov 9;24:101986. doi: 10.1016/j.fochx.2024.101986 (PMC11605458; doi:10.1016/j.fochx.2024.101986)
Supplement: Supplementary material 1 — Supplemental Table S1: Information for the identified carotenoids degradation related ZjNCED, starch degradation related ZjBAM, sucrose biosynthesis related ZjSPS, flavonoids biosynthesis related ZjPAL and ZjCHS, and anthocyanin biosynthesis related ZjDFR in winter jujube fruits. [file mmc1.docx]

**Table S1** Information for the identified carotenoids degradation related *ZjNCED*, starch degradation related *ZjBAM*, sucrose biosynthesis related *ZjSPS*, flavonoids biosynthesis related *ZjPAL* and *ZjCHS*, and anthocyanin biosynthesis related *ZjDFR* in winter jujube fruits.

| **Gene ID** | **Homologous gene ID** | **DZ gene name** | **Identity (%)** | **Bit score** |
| --- | --- | --- | --- | --- |
| gene-LOC107420293 | AT5G42800.1 | *ZjDFR* | 72.595 | 533 |
| gene-LOC107409395 | AT4G19170.1 | *ZjNCED* | 70.251 | 814 |
| gene-LOC107413687 | AT3G53260.1 | *ZjPAL* | 84.081 | 1232 |
| gene-LOC107422581 | AT5G13930.1 | *ZjCHS* | 86.269 | 696 |
| gene-LOC107431507 | AT5G18670.1 | *ZjBAM* | 60.589 | 626 |
| gene-LOC125420704 | AT5G20280.1 | *ZjSPS* | 78.997 | 1710 |
