# Supplementary material for: Piriformospora indica culture filtrate application adds brilliance to the promoting effects of facility warming on winter jujube fruit ripening
Source: Food Chem X. 2024 Nov 9;24:101986. doi: 10.1016/j.fochx.2024.101986 (PMC11605458; doi:10.1016/j.fochx.2024.101986)
Supplement: Supplementary material 2 — Supplemental Table S2: Information for the primers used in this study. [file mmc2.docx]

**Supplemental Table S2** Information for the primers used in this study.

| **Gene name** | **Forward primer (5’→3’)** | **Reverse primer (5’→3’)** | **Annealing temperature (℃)** | **Target length (bp)** |
| --- | --- | --- | --- | --- |
| *Actin* | AGCCTTCCTGCCAACGAGT | TTGCTTCTCACCCTTGATGC | 57.7 | 125 |
| *ZjNCED* | TGCCGTCATAAACCAGTCCT | CTCCTCCTCCTCGTGATGAC | 56.8 | 220 |
| *ZjPAL* | ACTCTCGGATACAGCTCGTG | GCTCTTGTTGCTGTGTGTGA | 56.2 | 231 |
| *ZjCHS* | TTTTGGGCCTACGTCCTTCT | GGAAAGTAACCGCGGTGATC | 56.3 | 156 |
| *ZjBAM* | TGCCTGGAATGGACTTAGCA | CCATGTTTTCTGCAGGCTGT | 56.2 | 100 |
| *ZjSPS* | AGCAGCAAAGACAAAGGGTG | TAACCCATTCTGCCGACACT | 56.1 | 247 |
| *ZjDFR* | ATCGGATCATGGCTCGTCAT | ATCAGCCTTCCACAGTGTCA | 56.2 | 144 |
